# The role of plasma angiotensin II and angiotensin-converting enzyme 2 levels on prognosis and mortality in hypertensive patients with COVID-19

**DOI:** 10.2217/bmm-2021-0121

**Published:** 2021-10-27

**Authors:** Serap Biberoğlu, Afşin İpekci, İbrahim İkizceli, Fatih Çakmak, Yonca S Akdeniz, Altuğ Kanbakan, Dildar Konukoğlu, İbrahim M Bolayırlı, Şermin Börekçi, Seval Ürkmez, Seda Özkan

**Affiliations:** ^1^Department of Emergency Medicine, Cerrahpasa Faculty of Medicine, Istanbul University Cerrahpasa, Istanbul, Turkey; ^2^Department of Emergency Medicine, Yuksekova State Hospital, Hakkari, Turkey; ^3^Department of Medical Biochemistry, Cerrahpasa Faculty of Medicine, Istanbul University Cerrahpasa, Istanbul, Turkey; ^4^Department of Pulmonology, Cerrahpasa Faculty of Medicine, Istanbul University Cerrahpasa, Istanbul, Turkey; ^5^Department of Intensive Care (Anesthesia & Reanimation), Cerrahpasa Faculty of Medicine, Istanbul University Cerrahpasa, Istanbul, Turkey

**Keywords:** ACE2, ANG II, COVID-19, hypertension

## Abstract

**Introduction:** SARS-CoV-2 requires angiotensin-converting enzyme 2 (ACE2) to enter the cell. In our study, we aimed to investigate the role of angiotensin-converting enzyme 2 and angiotensin II plasma levels on prognosis and mortality in patients with isolated hypertension, patients with chronic diseases in addition to hypertension and patients with COVID-19 without comorbidities, in accordance with the use of renin–angiotensin–aldosterone system inhibitor. **Materials & methods:** In the study, patients diagnosed with COVID-19 were divided into three groups. Angiotensin II and ACE2 levels were compared by comorbidities, antihypertensive drugs used, intensive care hospitalization and termination of patients. The relationship between angiotensin II and ACE2 levels and service and intensive care times was investigated. **Findings:** A total of 218 patients were enrolled in our study, including 68 patients diagnosed with COVID-19 without comorbidities, 33 patients diagnosed with isolated hypertension and 117 patients with other chronic diseases in addition to hypertension. There was no statistically significant difference between the comorbid disease groups between angiotensin II and ACE2 levels of the patients enrolled in the study. The rate of patients admitted to the intensive care unit was 17.9%, and the mortality rate was 11.5%. **Results:** In our study, we did not obtain significant findings regarding angiotensin II and ACE2 levels on presentation that can be used in prognosis and mortality of COVID-19 patients and development of future treatment methods.

Cases of pneumonia of unknown etiology were reported by the World Health Organization in December 2019 from Wuhan, Hubei Province, China, where fever, shortness of breath and radiological finding of bilateral pulmonary pneumonic infiltration were seen in these cases. It was called SARS-CoV-2 or 2019-nCoV due to its close similarity to  SARS-CoV. It was reported that mortality was generally seen in patients with advanced age or in patients with accompanying systemic diseases (hypertension, diabetes, cardiovascular disease, cancer, chronic lung diseases) [1,2].

Angiotensin-converting enzyme 2 (ACE2) is an enzyme within the renin–angiotensin–aldosterone system (RAAS), which is expressed on the surface of type 2 alveolar epithelial cells in the lungs and in cells in many other tissues. It also acts as a receptor for the SARS-CoV-2 spike protein through which the virus enters the host cells. The affinity of SARS-CoV-2 for ACE2 is 10–20-times higher than that of SARS-CoV, which may explain its higher transmissibility. Spike protein's binding to ACE2, accompanied by the proteolytic division of transmembrane serine protease 2 (TMPRSS2) and ACE2, facilitates the introduction of the virus into cells, viral replication and intercellular transition [3–5].

ACE produces Ang II from Ang I, ACE2 generates Ang (1–9) from Ang I or Ang (1–7) from Ang II, respectively. Ang (1–7) possesses vasodilator, antioxidant and anti-inflammatory properties that, upon binding to the Mas receptor broadly, shifts the balance from vasoconstriction with Ang II to vasodilation with Mas receptor activation in the affected vascular bed [6].

ACE2 converts angiotensin II (ANG II) to ANG (1–7), which acts on the Mas receptor, and it is expressed in various cell lines in many tissues associated with cardiovascular disease (including type 2 alveolar epithelial cells) [3]. The fact that ACE–ANG II activity is greater than the activity of ACE2-Ang- (1–7) indicates that SARS-CoV-2 may lead to acute lung injury [7]. Proteolytic cleavage and viral entry after binding of the SARS-CoV-2 spike protein to ACE2 is suggested to suppress ACE2 expression. ANG (1–7) levels further decrease as ACE converts ANG (1–7) to less biologically active peptides [7,8]. Furthermore, soluble form of ACE2 obstructs SARS-CoV-2 from binding to membrane-anchored ACE2 in plasma membrane. An increased amount of soluble ACE2 and expression induced due to RAS inhibitors could be advantageous for protecting lungs and other organ injury, but not infection with SARS-CoV-2 [9].

Although the effects of such drugs as ACE inhibitors (ACEIs) and angiotensin-receptor blockers (ARB) on ACE2 in patients with COVID-19 remain unclear, previous findings led to increased concern that ACEIs and ARBs may increase (or decrease) mortality in patients with COVID-19 [10,11]. Therefore, according to the use of RAAS inhibitors, we aimed to investigate the role of ACE2 and ANG II plasma levels on disease prognosis and mortality in patients with isolated hypertension, in patients with chronic diseases in addition to hypertension and in patients with COVID-19 without comorbidities.

## Materials & methods

Our prospective observational study was conducted between 10 June and 1 August 2020 after approval of the Ethics Committee of Istanbul University–Cerrahpaşa, Cerrahpaşa Faculty of Medicine, dated 6 May 2020 and numbered 68359.

Inclusion criteria included patients who were older than 18 years and were hospitalized after being diagnosed with COVID-19 infection in the emergency department (ED) were included in the study. COVID-19 infection was diagnosed in the ED by using nasal swap samples for PCR test and CT scan. Exclusion criteria included negative PCR test results for COVID-19 in pneumonia patients, negative CT scan with positive PCR test results, pregnant women and patients who were transferred another hospital during follow-up. The first endpoint was intensive care unit (ICU) admission, and second endpoint was the mortality.

Peripheral venous blood samples for plasma ANG II and ACE2 levels were collected from the patients at the time of admission to the ED. Patients’ presenting symptoms, vital signs, chronic diseases, blood parameters and medications were recorded. The patients were followed up during their hospitalization. The patients’ hospitalization periods, admission to the ICU and its duration and mortality or discharge status were recorded. Written consent was obtained from the patients who wanted to participate in the study.

In the study, patients diagnosed with COVID-19 were divided into three groups by the presence of comorbidities. Group 1 was composed of patients diagnosed with COVID-19 without any comorbidities or chronic drug use, whereas Group 2 included patients diagnosed with COVID-19 with only hypertensive (HT) disease; Group 3 included patients diagnosed with COVID-19 with one or more other chronic diseases in addition to hypertension. In our study, other chronic comorbidities in patients diagnosed with COVID-19 were diabetes mellitus, chronic obstructive pulmonary disease (COPD), chronic renal failure (CRF), malignancies, cerebrovascular disease (CVO), rheumatic diseases, thyroid diseases and coronary artery diseases. Patients diagnosed with hypertension were divided into groups by the antihypertensive drugs they used (those using only ACEIs, only ARBs, only calcium channel blockers or only beta blockers and those on multiple antihypertensive drugs). ANG II and ACE2 levels were compared in the disease groups, in the antihypertensive drug groups, by the intensive care unit (ICU) admission and termination. The relationship between ANG II and ACE2 levels and the length of hospital stay in the service and ICU was investigated.

Peripheral venous blood samples collected from the patients were centrifuged at 3500 r.p.m. for 10 min, and serum samples were portioned and stored frozen at -80°C until analysis. Serum ANG II and ACE2 levels were determined using ELISA. Competitive ELISA analysis (Elabscience Biotechnology Inc., catalog no. E-EL-H0326, TX, USA) was used for ANG II and Sandwich ELISA (Elabscience Biotechnology Inc., catalog no. E-EL-H0281) for ACE2 levels. The method was applied pursuant to the manufacturer’s recommendations. Day-to-day and within-run coefficient of variation values were 10 and 95% for ANG II and 10.4 and 11.5% for ACE2, respectively. Results were provided in pg/ml for ANG II and ng/ml for ACE2 (CH3). Sensitivity of the ANG II kit was 18.75 and 18.75 pg/ml for ACE2.

Venous blood samples were placed in EDTA containing tubes (BD vacutainer, ref. no. 367836, NJ, USA) for blood count parameters; anticoagulant-free gel tubes (Vacuette, Greiner-Bio, ref. no. 455071, Kremsmünster, Austria) for aspartate aminotransferase (AST), alanine aminotransferase (ALT), creatinine (Cr), lactate dehydrogenase (LDH), C-reactive protein (CRP), ferritin (FER) and procalcitonin (PCT), and tubes containing sodium citrate as anticoagulant (Vacuette, Greiner-Bio, ref. no. 454322, Kremsmünster, Austria) for D-dimer and fibrinogen analysis. For biochemistry analysis, blood samples were centrifuged at 3500 r.p.m. for 10 min. All routine analyzes were carried out within 2 h at the latest without waiting in the laboratory, and blood gas analyses were performed within 20 min.

Complete blood count and determination of leukocyte subgroups was performed using Beckman Coulter LH 780 analyzer (CA, USA) and AST, ALT and LDH were determined on Roche Cobas 8000 analyzer (Roche Diagnostics, Basel, Switzerland) using enzymatic kinetic method, Cr colorimetric photometric method, and CRP immunoturbidimetric methods. Sysmex CS2500 hemagglutinin analyzer (Kobe, Japan) was used for D-dimer and fibrinogen analysis. Ferritin and PCT analyses were performed using the electrochemiluminescence method on the Roche Cobas 8000 modular analyzer (Basel, Switzerland).

### Statistical analyses

Statistical analysis of the data was performed using SPSS 20 software. Kolmogorov-Smirnov test was used in the normality analysis of the data. Frequency (n), percent (%), median, minimum and maximum (min–max) values were used for the purpose of descriptive statistics. Mann–Whitney U test was used for comparison of two independent groups without a normal distribution. Chi-square test and Fisher’s exact test were used to compare the frequency data of independent groups. The Kruskal–Wallis test was used to compare more than two independent groups without a normal distribution. Spearman correlation test was used for correlation analysis of the data. p < 0.05 was considered statistically significant.

## Results

A total of 218 patients that met the criteria were included in our study, of which 56.4% of the patients were male and 45.4% were female. The most common symptom in the patients was cough (63.8%), followed by weakness (54.6%), fever (50%) and shortness of breath (44%), respectively. Although 17.4% of the patients did not require respiratory support, nasal cannula (33%) and nasal mask (23.4%) were the most frequently used respiratory support methods. The ICU admission rate was 17.9%, and the mortality rate was 11.5% (Table 1).

**Table 1.** Patient demographic data.

Characteristics	n	%
**Gender**		
– Male	119	54.6
– Female	99	45.4
**Presenting symptoms**		
– Fever	109	50
– Weakness	119	54.6
– Cough	139	63.8
– Shortness of breath	96	44.0
– Sore throat	16	7.3
– Diarrhea	11	5
– Lose of smell and taste sense	10	4.6
– Headache	14	6.4
– Myalgia	84	38.5
– Other symptoms	37	17.0
**Antihypertensive drugs used**		
– ACEI	51	23.4
– ARB	59	27.1
– β-blockers	72	33.0
– Diuretics	81	37.2
– Calcium channel blockers	58	26.6
Intensive care unit admission	39	17.9
Mortality	25	11.5

ACEI: Angiotensin-converting enzyme inhibitor; ARB: Angiotensin-receptor blockers.

The median age of the patients was significantly higher in Group 2 and 3 than in Group 1. Upon review of the vital signs of the patients in our study by the groups, systolic blood pressure (SBP), diastolic blood pressure (DBP), and respiratory rate (RR) were significantly higher in Group 2 and 3 when compared with Group 1. Whereas, sPO2 values were significantly lower in Group 3 when compared with the other groups. In terms of laboratory parameters, a significant difference was found in white blood cell, CRP, and D-dimer values between the groups, with the highest values seen in Group 3. ICU admission and mortality rates were highest in Group 3 with 24.8% and 17.1%, respectively (Table 2).

**Table 2.** Comparison of vital signs, laboratory parameters, ANG II, and ACE2 levels of comorbid disease groups.

Characteristics	Group 1, n = 68, 31.2%	Group 2, n = 33, 15.1%	Group 3, n = 117, 53.7%	p-value
Age (year)	51 (21–84)	67 (44–92)	65 (36–93)	**<0.001**
ICU, n (%)	6 (8,8%)	4 (12.1%)	29 (24.8%)	**0.015**
Mortality, n (%)	65 (95.6%)	31 (93.9%)	97 (82.9%)	**0.019**
**Vital signs, median (min–max)**				
Fever (°C)	37 (36–39)	37 (36–39)	36.8 (35.40–40)	0.375
SBP (mmHg)	120 (87–160)	130 (110–200)	130 (75–180)	**<0.001**
DBP (mmHg)	70 (50–110)	80 (56–120)	80 (44–100)	**0.003**
RR/minute	22 (16–38)	22 (18–30)	24 (18–40)	**<0.001**
sPO_2_ (%)	96 (86–99)	95 (88–98)	93 (54–99)	**<0.001**
**Laboratory parameters**, **median (min–max)**				
WBC (10^3^/μl)	5.65 (2.50–14.70)	6.70 (3.20–13.30)	7.40 (1.90–32.60)	**<0.001**
Lymphocyte (10^3^/μl)	1.25 (0.2–6.1)	1.2 (0.5–2.9)	1.4 (0.1–5.6)	0.766
CRP (mg/l)	26.4 (0.47–315)	30 (1.57–296.82)	57 (1.14–308)	**0.006**
Ferritin (ng/ml)	210 (14–1713)	399 (16–1266)	200.50 (26–3845)	0.160
Fibrinogen (mg/dl)	424 (120–900)	464 (260–875)	464 (150–900)	0.326
D-dimer (mg/l)	0.61 (0.19–51)	0.70 (0.19–11.80)	1.19 (0.19–80)	**<0.001**
ACE2 (ng/ml)	90.25 (39.44–194.82)	85.80 (36.47–200.37)	90.54 (25.26–192.64)	0.371
ANGII (pg/ml)	448.05 (143.56–1466.03)	358.43 (150.55–1551.62)	449.55 (101.30–2997.91)	0.314

The bolded p-values indicate statistically significant.

ACE: Angiotensin-converting enzyme; ANG: Angiotensin; CRP: C-reactive protein; DBP: Diastolic blood pressure; RR: Respiratory rate; SBP: Systolic blood pressure; WBC: White blood cell.

The plasma ANG II–ACE2 median levels were 448.05 pg/ml and 90.25 ng/ml in Group 1, 358.43 pg/ml and 85.80 ng/ml in Group 2 and 449.55 pg/ml and 90.54 ng/ml in Group 3. Although the values in the isolated HT group (Group 2) were lower than the others, this was not statistically significant. Likewise, although both ANG II and ACE2 were found to be higher in Group 3, this was also not statistically significant (Table 2).

There was no statistically significant difference in ANG II and ACE2 levels of the patients when comparing ICU admission and termination (Table 3). Although the serum ANG II levels of the patients who died were lower than those who survived, this was not statistically significant (Table 3).

**Table 3.** Comparison of patients’ vital signs, laboratory parameters, ANG II and ACE2 levels of patients by ICU admission and mortality.

	ICU admission, n (%)			Mortality		
	No, n = 179, 82.1%	Yes, n = 39, 17.9%	p-value	Survivor, n = 193, 88.5%	Nonsurvivor, n = 25, 11.5%	p-value
Age (year)	60 (21–93)	70 (30–94)	**0.001**	60 (21–93)	70 (55–90)	**0.001**
**Vital signs, median (min–max)**						
Fever (°C)	36.90 (35.70–40)	37.50 (35.4–39.10)	0.076	37 (35.70–40)	37.60 (35.4–39.1)	**0.040**
SBP (mmHg)	120 (80–200)	136 (75–180)	0.040	120 (80–200)	138 (75–180)	0.156
DBP (mmHg)	75 (50–120)	70 (44–100)	0.473	75 (44–120)	70 (45–100)	0.230
RR/minute	22 (16–40)	28 (18–40)	**<0.001**	22 (16–40)	28 (18–40)	**<0.001**
sPO_2_ (%)	95 (84–99)	89 (54–97)	**<0.001**	95 (70–99)	87 (54–97)	**<0.001**
**Laboratory parameters, median (min–max)**						
WBC (10^3^/μl)	6.52 (2.4–25.1)	7.81 (1.9–32.6)	0.032	6.52 (2.4–25.1)	8.10 (1.9–32.6)	**0.039**
Lymphocyte (10^3^/μl)	1.40 (0.3–6.1)	0.80 (0.1–5.6)	**<0.001**	1.40 (0.3–6.1)	0.70 (0.1–3.2)	**<0.001**
CRP (mg/l)	31.10 (0.47–315)	90 (3–297)	**<0.001**	31.48 (0.47–315)	104 (3–297)	**<0.001**
Ferritin (ng/ml)	198 (14–2000)	599 (34–3845)	**<0.001**	205 (14–2000)	559 (34–3845)	**<0.001**
Fibrinogen (mg/dl)	450 (120–900)	482 (150–900)	0.075	450 (120–900)	516 (150–900)	**0.023**
D-dimer (mg/l)	0.73 (0.19–51)	2.19 (0.20–80)	**<0.001**	0.75 (0.19–51)	3.70 (0.29–80)	**<0.001**
ACE2 (ng/ml)	88.72 (39.44–200.37)	90.31 (25.26–192.64)	0.857	90.04 (36.47–200.37)	87.41 (25.26–192.64)	0.940
AngII (pg/ml)	446.06 (101.30–2997.91)	430.30 (176.87–1455.96)	0.263	449.55 (101.30–2997.91)	367.60 (176.87–1455.96)	0.095

ACE: Angiotensin-converting enzyme; ANG: Angiotensin; CRP: C-reactive protein; DBP: Diastolic blood pressure; RR: Respiratory rate; SBP: Systolic blood pressure; WBC: White blood cell.

The bolded p-values indicate statistically significant.

Analyses of the ANG II and ACE2 levels of the patients by the types and use of antihypertensive drugs showed that the highest ACE2 level was in patients using combined antihypertensive drugs and the highest ANG II level was found in the groups using only ACEIs or only ARBs, but this was not statistically significant (Table 4). ICU hospitalization rate was statistically significantly higher in patients using combined antihypertensive drugs, but mortality was not statistically significant, although it was found to be higher in this group (Table 4).

**Table 4.** ANG II and ACE2 levels, other datas in patients according to antihypertensive drug uses.

Characteristics	No anti-HT	ACEI or ARB	Other anti-HT	Combined anti-HT	p-value
Age (year)	51 (21–84)	64 (48–92)	63.50 (36–93)	70 (42–88)	**<0.001**
HT group	-	12	9	12	0.774
HT/comorbid group	-	35	33	49	
ICU	6	7	6	20	**0.003**
Mortality	3	5	5	12	0.056
SBP (mmHg)	120 (87–160)	120 (75–170)	130 (80–200)	130 (80–180)	**<0.001**
DBP (mmHg)	70 (50–110)	75 (45–110)	80 (56–120)	80 (44–96)	**0.020**
Lymphocyte (10^3^/μl)	1.25 (0.2–6.1)	1.40 (0.4–4)	1.40 (0.1–3.3)	1.30 (0.3–5.6)	0.836
CRP (mg/l)	26.40 (0.47–315)	31.48 (1.19–220)	57.75 (1.71–254)	45.00 (1.14–308)	0.057
Ferritin (ng/ml)	210 (14–1713)	331 (26–3845)	204.50 (29–2000)	248 (16–3786)	0.381
D-dimer (mg/l)	0.61 (0.19–51)	0.95 (0.19–35.65)	1.11 (0.28–35.20)	1.22 (0.19–80)	**0.002**
Fibrinogen (mg/dl)	424 (120–900)	435 (150–754)	464 (268–875)	495 (173–900)	0.253
AngII (pg/ml)	448.05 (143.56–1466)	499.33 (178.95–2725.80)	384.17 (150.55–2997.91)	434.70 (101.30–2560)	0.417
ACE2 (ng/ml)	90.25 (39.44–194.82)	86.87 (25.26–200.37)	85.78 (55.99–189.96)	94.49 (36.47–173.47)	0.197

ACE: Angiotensin-converting enzyme; ACEI: Angiotensin-converting enzyme inhibitor; ANG: Angiotensin; ARB: Angiotensin-receptor blockers; CRP: C-reactive protein; DBP: Diastolic blood pressure; HT: Hypertensive treatment; RR: Respiratory rate; SBP: Systolic blood pressure; WBC: White blood cell. The bolded p-values indicate statistically significant.

## Discussion

ACE2, one of the key RAAS-modulating enzymes, has received increased attention during the pandemic period because it not only converts ANG II to ANG (1–7) but also acts as the cellular entry receptor for SARS-CoV-2. Nevertheless, it remains unclear how RAAS activity, especially ACE2, takes place in COVID-19 and how the RAAS activity changes in patients on ACEI/ARB treatment. In this study, we analyzed the effect of plasma ACE2 and ANG II levels on the prognosis and mortality of patients with COVID-19. Our study provided important information on potential targets in SARS-CoV-2 infection for those with chronic diseases and chronic antihypertensive drug use.

In our study on patients with COVID-19, no statistically significant difference was found in ANG II and ACE2 levels of HT patients with isolated ACEI and ARB use and those without chronic drug use.

A study by Sama *et al.,* is the first major study examining the relationship between ACE2 plasma concentrations and the use of RAAS blockers in patients with cardiovascular disease [12]. In contrast to the previous reports [13–16], in this study ACEIs and ARBs, were associated with increased plasma concentrations of ACE2, consistent with our findings. In fact, use of ACEIs and ARBs, although these findings were not repeated in the index cohort, predicted lower ACE concentrations in verification cohort [12]. As a whole, these data do not support the discontinuation of ACEIs or ARBs in patients at risk for SARS-CoV-2 infection.

Kintscher *et al.,* found that there was no significant difference between healthy individuals and patients with COVID-19 by plasma ANG I + II, ANG II/ANG I and ACE2 activity. These data suggest that there is no increased RAAS activity level in patients with COVID-19, and in particular, COVID-19 originated alternative RAS activation as potentially mediated by circulating ACE2 is not a typical feature. ACE2 activity was significantly higher in patients with COVID-19 using ACEIs compared with patients with COVID-19 who were not receiving ACEI/ARB therapy. They found that the use of ARBs in COVID-19 did not significantly affect ACE2 activity [17]. In our study, there was no difference in ACE2 and ANG II levels between patients without chronic drug use and HT patients using ACEIs or ARBs. ANG II level was found to be high in HT patients using only ARB, but it was not statistically significant. Plasma ACE2 level was higher in the group using multiple antihypertensive drugs, but there was no statistical significance.

Yang and Wang *et al.,* found that patients with COVID-19 with comorbidities would more likely develop critical disease compared with those without comorbidities [18,19]. Our study associated the presence of comorbidity (Group 3) with mortality and was consistent with the aforementioned study. Especially COPD, CRF, heart failure and presence of malignancy were statistically significantly higher in patients who died. Serum ANG II levels of the patients who died were significantly lower than those who survived, but there was no statistical significance. Also, Ozkan *et al.* found that no statistically significant difference was between the ANG II levels in the blood samples of the patients with and without comorbid disease, which were taken at the time of admission to the ED [20].

Meng *et al.,* compared the clinical and laboratory findings of the patients who used ACEIs and ARBs and patients diagnosed with COVID-19 who were on other antihypertensives and found no statistical significance [21]. In our study, there was no statistically significant difference in laboratory parameters and mortality in HT patients with COVID 19 using only ACEIs and only ARBs compared with those using other antihypertensives and multiple antihypertensives. This supported studies that found that the use of ACEIs and ARBs did not affect the clinical outcome and prognosis of the disease.

A retrospective cohort study by Fosbol *et al.* found that the use of ACEIs or ARBs among 4480 patients diagnosed with COVID-19 was not significantly associated with changes in mortality. It was concluded that the findings did not support the discontinuation of ACEI/ARB drugs indicated in HT patients during the COVID-19 pandemic [22]. In our study, there was no difference in mortality between the patients using ACEI/ARB and those who did not, which was consistent with the preceding study.

In their study Reynolds *et al.,* investigated the association of the use of ACEIs, ARBs, β-blockers, calcium channel blockers or thiazide diuretics with the likelihood of positive or negative COVID-19 test results. In patients with a positive PCR test, the analysis of prognosis of disease (intensive care, mechanical ventilation or death) suggested that there was no association with the use of single antihypertensive drug and an increase in the likelihood of a positive PCR test [23]. It was also not associated with a significant increase in the risk of development of serious disease in patients with positive PCR test among these groups using antihypertensive drugs [23].

Sardu *et al.,* found that antihypertensive drugs did not affect the prognosis in patients with COVID-19. In that study, the authors analyzed the effects of ACEIs versus ARBs versus calcium channel blockers drugs on clinical outcomes as mechanical ventilation, ICU admissions, heart injuries and death in patients with hypertension hospitalized for COVID-19. In addition, they did not assay ACE2 expression in patients with HT [24]. Also in our study, the ICU hospitalization rate was statistically significantly higher in patients using combined antihypertensive drugs, but mortality was not statistically significant, although it was found to be higher in this group.

A study by Wang *et al.,* on patients using ACEI/ARB and non-ACEI/ARB did not find a statistically significant difference in death rate, cumulative survival rate, duration of hospitalization/ICU admission, clinical outcomes or adverse events [25]. The results regarding mortality and prognosis were consistent with our study. Therefore, the fact that there was no statistical significance with regard to mortality when compared with the antihypertensive drug used by the patients and that there was no statistically significant difference upon comparison of both ACE2 and ANG II levels in our study suggests that ACEI/ARB use did not affect prognosis. Comparatively, there was no significant difference in ACE2 and ANG II plasma levels of 218 patients divided into three groups in our study. Although plasma ACE2 and ANG II levels were lower in Group 2 (isolated HT patients), there was no statistical significance. Therefore, we suggest that there is no evidence that ACEIs/ARBs should be discontinued in the COVID-19 pandemic.

## Conclusion

Our data suggest that serum ANG II and ACE2 levels of patients with COVID-19 are not affected by hypertension and ACEI or ARB use. The fact that RAAS is dependent on many variables in the human body may cause changes in ANG II and ACE2 levels. However, we think that further studies are necessary to be able to use the plasma levels of ANG II and ACE2 in predicting the prognosis of patients with COVID-19 and developing future treatment methods.

**Summary points**

- In this study, the role of angiotensin-converting enzyme (ACE) and angiotensin II (ANG II) plasma levels on disease prognosis and mortality was investigated in isolated-hypertensive (HT) patients, patients with chronic disease in addition to hypertension and COVID-19 patients without comorbidity.
- There was no statistically significant difference in ANG II and ACE2 levels of the patients when comparing ICU admission and termination.
- In terms of laboratory parameters, a significant difference was found in white blood cell, CRP, and D-dimer values between the groups, with the highest values seen in Group 3 (included patients diagnosed with COVID-19 with one or more other chronic diseases in addition to hypertension).
- Analyses of the ANG II and ACE2 levels of the patients by the types and use of antihypertensive drugs showed that the highest ACE2 level was in patients using combined antihypertensive drugs and the highest ANG II level was found in the groups using only ACEIs or only ARBs, but it was not statistically significant.
- ICU hospitalization rate was statistically significantly higher in patients using combined antihypertensive drugs, but mortality was not statistically significant, although it was found to be higher in this group.
- Serum ACE2 and ANG II levels were lower in Group 2 (isolated HT patients), there was no statistical significance.
- There is no evidence yet that ACEIs/ARBs should be discontinued in the COVID-19 pandemic.
- Serum ANG II and ACE2 levels of patients with COVID-19 are not affected by hypertension and ACEI or ARB use.

## References

[1] Rehberi TC, Sağlık Bakanlığı, Halk Sağlığı Covid-19 (SARS-CoV-2 Enfeksiyonu) (2020). https://covid19bilgi.saglik.gov.tr/depo/rehberler/COVID-19_Rehberi.pdf

[2] Hoffmann M, Kleine-Weber H, Schroeder S SARS-CoV-2 cell entry depends on ACE2 and TMPRSS2 and is blocked by a clinically proven protease inhibitor. Cell 181(2), 271–280 (2020). 32142651 10.1016/j.cell.2020.02.052 PMC7102627

[3] Sparks MA, Crowley SD, Gurley SB Classical renin-angiotensin system in kidney physiology. Compr. Physiol. 4(3), 1201–1228 (2014). 24944035 10.1002/cphy.c130040 PMC4137912

[4] Matarese A, Gambardella J, Sardu C, Santulli G. miR-98 Regulates TMPRSS2 expression in human endothelial cells: key Implications for COVID-19. Biomedicines 8(11), 462 (2020). 10.3390/biomedicines8110462 PMC7693865 33143053

[5] Zhang T, Zhong S, Cao W. COVID-19 and comorbid hypertension: is ACE2 the culprit? Prehosp. Disaster Med. 35(6), 700–702 (2020). 32838828 10.1017/S1049023X20001090 PMC7471567

[6] Bosso M, Thanaraj TA, Abu-Farha M The two faces of ACE2: the role of ACE2 receptor and its polymorphisms in hypertension and COVID-19. Molecular Ther. Methods Clin. Dev. 18, 321–327 (2020). 10.1016/j.omtm.2020.06.017 PMC7314689 32665962

[7] Kuba K, Imai Y, Rao S A crucial role of angiotensin converting enzyme 2 (ACE2) in SARS coronavirus-induced lung injury. Nat. Med. 11, 875–874 (2005). 16007097 10.1038/nm1267 PMC7095783

[8] Ni W, Yang X, Yang D Role of angiotensin-converting enzyme 2 (ACE2) in COVID-19. Crit. Care. 24(1), 422 (2020). 32660650 10.1186/s13054-020-03120-0 PMC7356137

[9] Kumar P, Sah AK, Tripathi G Role of ACE2 receptor and the landscape of treatment options from convalescent plasma therapy to the drug repurposing in COVID-19. Mol. Cell Biochem. 476(2), 553–521 (2021). 33029696 10.1007/s11010-020-03924-2 PMC7539757

[10] Vaduganathan M, Vardeny O, Michel T Renin–angiotensin–aldosterone system inhibitors in patients with Covid-19. N. Engl. J. Med. 382, 1653–6 (2020). 32227760 10.1056/NEJMsr2005760 PMC7121452

[11] Patel AB, Verma A. COVID-19 and angiotensin-converting enzyme inhibitors and angiotensin receptor blockers: what is the evidence? JAMA. 323(18), 1769–1761 (2020). 32208485 10.1001/jama.2020.4812

[12] Sama IE, Ravera A, Santema BT Circulating plasma concentrations of angiotensin-converting enzyme 2 in men and women with heart failure and effects of renin-angiotensin-aldosterone inhibitors. Eur Heart J. 41(19), 1810–1817 (2020). 32388565 10.1093/eurheartj/ehaa373 PMC7239195

[13] Diaz JH. Hypothesis: angiotensin-converting enzyme inhibitors and angiotensin receptor blockers may increase the risk of severe COVID-19. J. Travel Med. 27(3), taaa041 (2020). 32186711 10.1093/jtm/taaa041 PMC7184445

[14] Kuster GM, Pfister O, Burkard T SARS-CoV2: should inhibitors of the renin–angiotensin system be withdrawn in patients with COVID-19? Eur. Heart J. 41(19), 1801–1803 (2020). 32196087 10.1093/eurheartj/ehaa235 PMC7184407

[15] Fang L, Karakiulakis G, Roth M. Are patients with hypertension and diabetes mellitus at increased risk for COVID-19 infection? Lancet Respir. Med. 8(4), e21 (2020). 32171062 10.1016/S2213-2600(20)30116-8 PMC7118626

[16] Zheng YY, Ma YT, Zhang JY, Xie X. COVID-19 and the cardiovascular system. Nat. Rev. Cardiol. 17(5), 259–251 (2020). 32139904 10.1038/s41569-020-0360-5 PMC7095524

[17] Kintscher U, Slagman A, Domenig O Plasma angiotensin peptide profiling and ACE2-activity in COVID-19 patients treated with pharmacological blockers of the renin angiotensin system. Hypertension 76(5), e34–2 (2020). 32851897 10.1161/HYPERTENSIONAHA.120.15841 PMC7480797

[18] Yang X, Yu Y, Xu J Clinical course and outcomes of critically ill patients with SARS-CoV-2 pneumonia in Wuhan, China: a single-centered, retrospective, observational study. Lancet Respir. Med. 8(5), 475–476 (2020). 32105632 10.1016/S2213-2600(20)30079-5 PMC7102538

[19] Wang D, Hu B, Hu C Clinical characteristics of 138 hospitalized patients with 2019 novel coronavirus-infected pneumonia in Wuhan, China. JAMA 323(11), 1061–1068 (2020). 32031570 10.1001/jama.2020.1585 PMC7042881

[20] Ozkan S, Cakmak F, Konukoglu D Efficacy of serum angiotensin II levels in prognosis of patients with coronavirus disease 2019. Crit. Care Med. 49(6), e613–e620 (2021). 33630767 10.1097/CCM.0000000000004967

[21] Meng J, Xiao G, Zhang J Renin-angiotensin system inhibitors improve the clinical outcomes of COVID-19 patients with hypertension. Emerg. Microbes Infect. 9(1), 757–753 (2020). 32228222 10.1080/22221751.2020.1746200 PMC7170368

[22] Fosbol EL, Butt JH, Ostergaard L Association of angiotensin-converting enzyme inhibitor or angiotensin receptor blocker use with COVID-19 diagnosis and mortality. JAMA 324(2), 168–169 (2020). 32558877 10.1001/jama.2020.11301 PMC7305566

[23] Reynolds HR, Adhikari S, Pulgarin C Renin-angiotensin-aldosterone system inhibitors and risk of Covid-19. N. Engl. J. Med. 382(25), 2441–2447 (2020). 32356628 10.1056/NEJMoa2008975 PMC7206932

[24] Sardu C, Maggi P, Messina V Could anti-hypertensive drug therapy affect the clinical prognosis of hypertensive patients with COVID-19 infection? Data from centers of Southern Italy. J. Am. Heart Assoc. 9(17), e016948 (2020). 32633594 10.1161/JAHA.120.016948 PMC7660768

[25] Wang Z, Zhang D, Wang S A retrospective study from 2 centers in China on the effects of continued use of angiotensin-converting enzyme inhibitors and angiotensin II receptor blockers in patients with hypertension and COVID-19. Med. Sci. Monit. 26, e926651 (2020). 32969367 10.12659/MSM.926651 PMC7523417

